# Click Reactions and Boronic Acids: Applications, Issues, and Potential Solutions

**DOI:** 10.3390/molecules15085768

**Published:** 2010-08-23

**Authors:** Chaofeng Dai, Yunfeng Cheng, Jianmei Cui, Binghe Wang

**Affiliations:** Department of Chemistry and Center for Biotechnology and Drug Design, Georgia State University, Atlanta, Georgia, 30303, USA

**Keywords:** Click chemistry, CuAAC, Boronic acids

## Abstract

Boronic acids have been widely used in a wide range of organic reactions, in the preparation of sensors for carbohydrates, and as potential pharmaceutical agents. With the growing importance of click reactions, inevitably they are also applied to the synthesis of compounds containing the boronic acid moiety. However, such applications have unique problems. Chief among them is the issue of copper-mediated boronic acid degradation in copper-assisted [2,3]-cycloadditions involving an alkyne and an azido compound as the starting materials. This review summarizes recent developments, analyzes potential issues, and discusses known as well as possible solutions.

## 1. Introduction

Boronic acids are very important in synthetic organic, materials, bioorganic, and medicinal chemistry as well as chemical biology. In organic chemistry, boronic acids are very important in Suzuki-Miyaura coupling [1], aromatic functionalization (such as amination) with a heteroatom-containing functional group [2], protection of diols [3], Diels-Alder reactions [4,5], asymmetric synthesis of amino acids [6], selective reduction of aldehydes [7], carboxylic acid activation [8,9,10], transition metal-catalyzed asymmetric conjugate additions of boronic acids [11,12], addition to carbonyl and imine derivatives [13,14,15], and as a template in organic synthesis [16]. In materials chemistry, boronic acids are important in crystal engineering [17,18], construction of polymers with reversible properties [19,20], building unique molecular architects [21,22,23,24], functionalization of nanostructures [25], separation and purification of glycosylated products [26,27] and feed-back controlled drug delivery (glucose) [28]. In bioorganic chemistry, boronic acid is a commonly used recognition moiety for the design and synthesis of sensors for carbohydrates [29], amino acids [30], amino alcohols [31,32], cyanides [33], fluoride [34] and α-hydroxy acids [35,36]. In medicinal chemistry, boronic acids are important for the preparation of inhibitors of hydrolytic enzymes [37] in boron neutron capture therapy (BNCT) [38], quorum sensing inhibition [39], antifungal agent development [40,41], and the inhibition of other enzymes [42]. Among all the biologically active boronic acids, bortezomib is an FDA-approved anticancer agent [43]. In chemical biology, boronic acids are used in the detection and sensing of peroxides [44,45], recognition and sensing of the tetraserine motif in protein [46], development of new MRI contrast agents [47,48], cell-surface carbohydrate biomarker recognition [49,50,51], and development of boronic acids-modified aptamers [52,53] and boronic acid-modified proteins for various sensing and purification applications [54].

Because of the tremendous importance of boronic acids, there is interest in finding ways to increase their structural diversity and to tether them to other scaffolds. Among all the methods available, copper-mediated Huisgen cycloaddition is one that requires mild conditions and easy to operate. Herein, we provide a brief overview of applications and issues in using Cu-assisted azide-alkyne cycloaddition (CuAAC) [55,56], commonly known as a click reaction, for increasing the structural diversity of boronic acid compounds. We will also touch upon the application of boronic acids in facilitating this cycloaddition reaction and how to minimize certain degradation problems.

## 2. The CuAAC Reaction-A Brief Overview

Click chemistry according to Sharpless’ definition [57] refers to reactions that are fast, versatile, of high yields, and require mild reaction conditions. In addition, the reaction products are easy to purify. Examples include the thiol-ene additions, oxime formation, nucleophilic additions to epoxides, Diels-Alder reactions, hetero-Diels-Alder reactions, and CuAAC [55,56]. The 1,3-dipolar cycloaddition between an azido compound and an alkyne was discovered in 1893 by Arthur Michael [58] and significantly developed in 1967 by Huisgen [59]. Without a metal catalyst, these cycloaddition reactions are relatively difficult, require high temperatures, and lack regioselectivity. In 2002, Meldal and Sharpless reported at almost the same time that Cu(I) was able to catalyze Huisgen [2+3] cycloadditions leading to fast and highly efficient azide–alkyne reactions with regio-chemistry control at room temperature in organic [60] or in polar media, such as *tert*-butyl alcohol, ethanol or pure water [55]. These important discoveries make the CuAAC reaction the most representative example of click reactions. As a result, in the literature, the term “click reaction” is often used to refer to the CuAAC reaction. Besides copper, ruthenium can also be used in such reactions, however, with a different regiochemistry outcome when compared with Cu(I) [61]. It has been proposed that during catalysis, copper binds to the terminal alkyne to form a π-complex, which leads to a substantially decreased p*K*a and allows for deprotonation in aqueous solution without additional added base [62]. These reactions are so robust that almost any source of solvated Cu(I) can be used as catalyst. However, the CuAAC reaction is not always without problems, due to potential oxidative side reactions. One commonly used solution is the use of Cu(II) in the presence of a reducing agent such as excessive sodium ascorbate. The use of a Cu(I) ligand can minimize side reactions such as oxidation and disproportionation. Another way of minimizing side reactions is the use of a chelating agent, such as tris-[(1-benzyl-1H-1,2,3-triazol-4-yl)methyl]amine (TBTA) [63] developed by the Sharpless lab. TBTA can accelerate the reaction by over 106-fold. The tetradentate binding by TBTA shields the Cu(I) from potential destabilizing interactions.

## 3. Click-Modification of Thymidine-5'-triphosphate (TTP) for DNA Incorporation

**Scheme 1:**
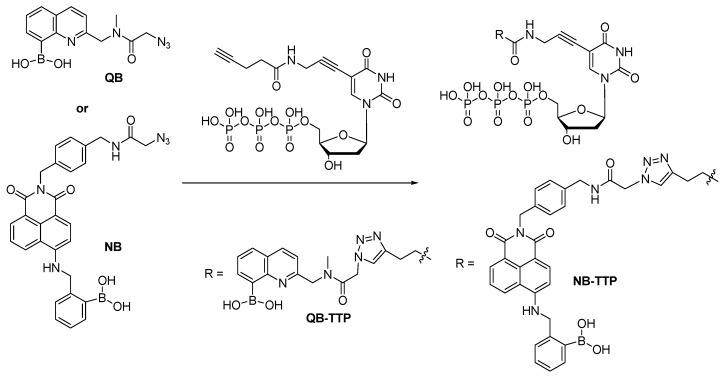
Synthesis of BQ-TTP and NB-TTP by CuAAC.

DNA is central to essentially to all life forms. Modified nucleosides/tides can be used as mechanistic probes for DNA polymerase and medicinal agents such as antiviral and anti-cancer drugs [64]. Besides, it has also been shown that side chain modified DNA can be used in the selection of functional aptamers with specific properties and high binding affinity [65]. In building side-chain functionalized DNA, there needs to be a way of either performing “site-specific” modification or synthesizing modified building blocks for incorporation chemically or enzymatically. For applications in aptamer selection, the evolutionary nature dictates that the incorporation needs to be enzymatic. Along this line, TTP analogs functionalized at the 5-position are known to be recognized by DNA polymerase and can be incorporated into DNA [65,66]. Therefore, boronic acid-modified TTP analogs were prepared for DNA aptamer selection and other applications. Specifically, an 8-quinolynylboronic acid-modified thymidine-5'-triphosphate (**QB-TTP**, Scheme 1) was first synthesized by the Wang lab [52]. In accomplishing the synthesis, there were two dilemmas. First, if the boronic acid moiety were introduced first, its hygroscopic nature would hinder the subsequent triphosphorylation, which required anhydrous conditions. On the other hand, if the triphosphate group were introduced first, the introduction of the boronic acid would also be very hard in using traditional chemistry such as amidation. Indeed, attempts using amidation and nucleophilic substitution all failed. Therefore, CuAAC was used in the synthesis of the very first 5-boronic acid-modified TTP, **QB-TTP** (Scheme 1). It was further demonstrated that **QB-TTP **could be effectively incorporated into DNA by enzymatic polymerization.

Recently, the Wang lab also prepared a new generation of boronic acid-modified TTP, **NB-TTP** (Scheme 1), by incorporating a naphthalenylboronic acid (**NB**) into TTP. The synthesis of **NB-TTP** followed the same procedure [53]. It is worth noting that during the synthesis of **NB-TTP**, it was found that the starting material **NB** could be precipitated out by methanol addition if the solution was acidic. Treatment with K_2_CO_3_ brought the boronic acid into solution. **NB-TTP** has the unique property of changing fluorescent properties upon sugar binding. In addition, the same fluorescent properties were preserved after DNA incorporation. For example, the fluorescent intensity of **NB-TTP ** increased upon the addition of model sugar, D-fructose with the *K*a of 73 M^-1^. After DNA incorporation, the fluorescent properties of NB-TTP-DNA were retained. The intensity increased by 1.5-fold upon addition of D-fructose (100 mM). This property is very important and can be used for future fluorescent DNA-based aptamer selection, DNA labeling as well as genomic DNA incorporation work. 

## 4. Click Reaction in the Preparation of Boronic Acid Fluorophores

**Scheme 2:**
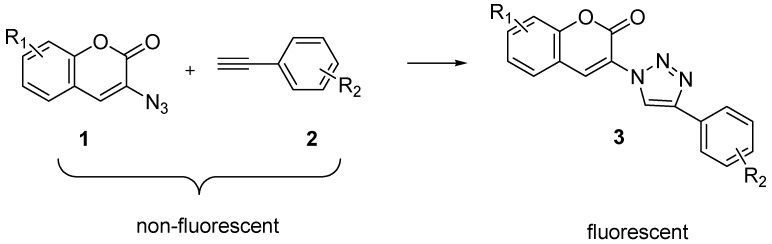
Synthesis of triazolylcoumarins by CuAAC.

Along the line of using the boronic acid group in preparing sensors, there are also interests in developing modular approaches for the construction of fluorescent boronic acids. Typically, the synthesis of boronic acid based sensors involves either direct attachment a boronic acids to a fluorophores or the attachment of a boronic acid to an amine to create boronic acid derivatives. Recently, CuAAC was used as a strategy for the preparation of a large number of fluorescent coumarin analogs. Triazole formation was used as a way to turn on coumarin fluorescence [67]. As shown in Scheme 2, 3-azidocoumarins **1** and terminal alkynes **2** are nonfluorescent. However, they afford a large library of intense fluorescent triazolylcoumarins **3** (λ_em_ = 388-521 nm) after click reaction. This reaction was used for the preparation of fluorescent boronic acids [42,68].

**Scheme 3:**
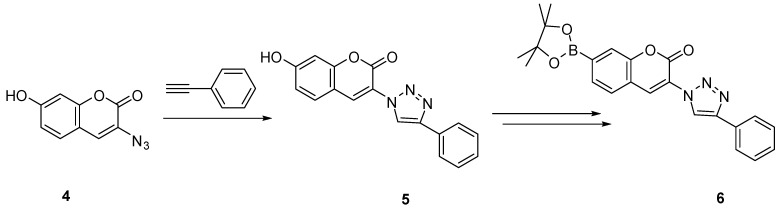
Synthesis of coumarin-based fluorescent probe **6 **by CuAAC.

Inspired by this, a new water-soluble “click” modified coumarin-based fluorescent boronic acid probe (**6**, Scheme 3) for hydrogen peroxide was designed and synthesized by the Wang lab [69]. The fluorescent intensity of probe **6 **at 5 µM increased by about 5-fold upon reaction for 120 min with 100 µM hydrogen peroxide. On the other hand, it showed much lower responses to other ROS species, such as hydroxyl radical, hypochlorite, *tert*-butyl hydroperoxide (TBHP) and *tert*-butoxy radical. By introducing the triazole ring through CuAAC, the excitation wavelength was increased by about 70 nm (λ_ex_ = 400 nm). Finally, the easy synthesis of probe **6** can also be adapted to include other functional groups for structural and spectroscopic diversity.

**Scheme 4:**
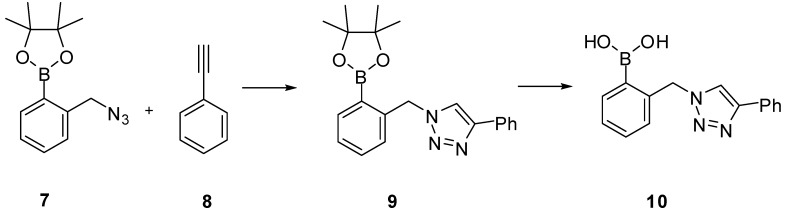
Synthesis of “click-flour” **10**.

In 2008, the James group reported a carbohydrate sensor (**10**, Scheme 4), which displayed fluorescent changes upon adding model saccharides [68]. Compound **10** was obtained by CuAAC and termed as a “click-fluor” because fluorescent changes were caused by the formation of 1,2,3-triazole moiety, instead of bringing the sensor and reporter units together. 

## 5. Synthesis of Alkynylarylboronic Acids for CuAAC

**Scheme 5:**
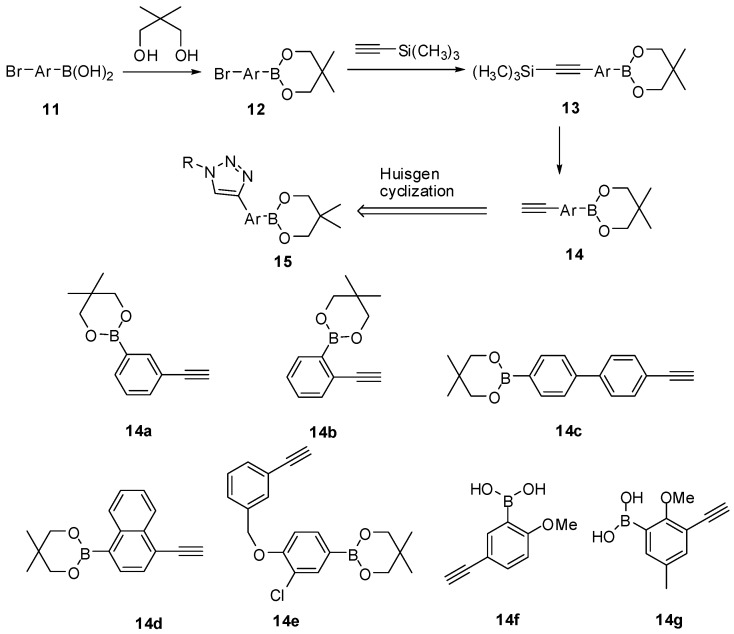
Synthesis of 2,2-dimethylpropane-1,3-diyl ethynylaryl boronates.

There are two ways of preparing boronic acids using CuAAC: by reacting an azidoarylboronic acid with a fluorophore bearing a terminal alkyne, or tethering a boronic acid containing a terminal alkyne with a fluorophore bearing an azido group. It is normally easy to prepare azidoarylboronic acids through bromination/halogenation and azido substitution as in the cases described in Scheme 1 and Scheme 4. However, the preparation of boronic acid containing a terminal alkyne is not straightforward. The Wang lab developed a practical, efficient and facile method for the synthesis of protected ethylnylaryl boronates (**14**, Scheme 5) from bromo-arylboronic acids [70]. The key step is the microwave-facilitated selective formation of trimethylsilylethynylaryl boronates **13** by Sonogashira reaction from the corresponding bromides. The use of microwave significantly improved the reaction yield and shortened the reaction time. The boronates **14 ** prepared can be subsequently used for the construction of boronic acids libraries with extended conjugations through CuAAC.

Besides the two ways of preparing boronic acids using CuAAC mentioned above, a reliable and operationally simple one-pot reaction for a one-carbon homologation of various aldehydes followed by a CuAAC was reported by Smietana and Vasseur in 2007 [71]. Compared to the other two ways mentioned, this reaction has a broad scope and can afford 1,2,3-triazoles in good to excellent yields from a variety of readily available aldehydes without the need for isolation of the alkyne intermediates. By taking advantage of this one-pot reaction, the authors prepared new boronic acid-based fluorescent sensors **16** in modest isolated yields (30~58%). This is the first preparation of fluorescent triazolylaryl boronates from commercially available formyl boronic acids. One more thing worth noting here is that this approach does not apply to *ortho* ethynyl boronates very well, compare to the *meta* and *para* analogues. The *ortho* ethynyl boronates gave a much lower yield (8% after purification), presumably due to steric constraints.

**Scheme 6:**
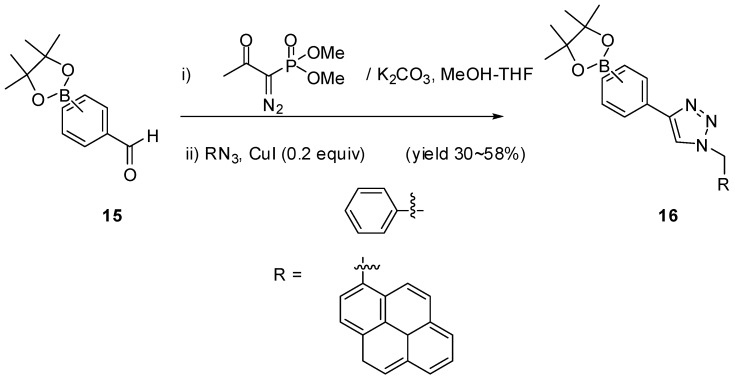
One pot synthesis of triazolyl boronates.

## 6. Bisamidoboronic Acid Preparation Using CuAAC

**Figure 1:**
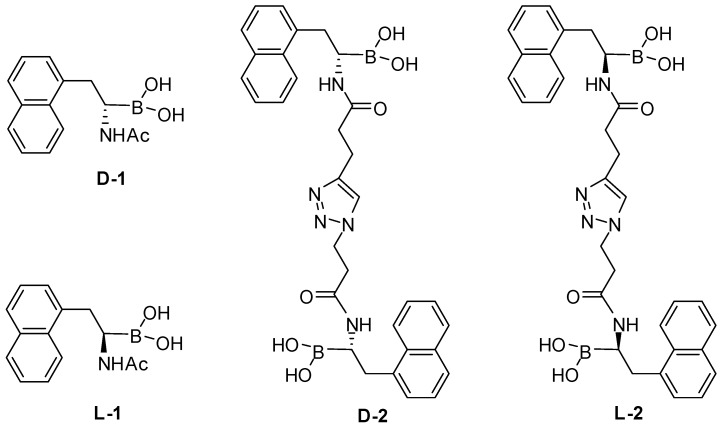
Enatiomeric pairs of α-amidoboronic acids and the corresponding bisboronic acids.

Due to their small size, high energy as well as narrow distribution of reactivity, alkyne and azido groups can be used as a pair of linkers to easily make bisboronic acid-based chemosensors. As a specific example, the Wang group recently reported two water soluble bis-α-amidoboronic acids (**D-2**, **L-2**, Figure 1) synthesized by using CuAAC [72]. Compared to their monoboronic acid counterparts (**D-1**, **L-1**, Figure 1), **D-2** and **L-2** showed significantly enhanced binding for oligosaccharides. For example, the *K*a of **L-2** with D-fructose is around 497 M^-1^, while the *K*a of **L-1** with D-fructose is 10-fold less at 46 M^-1^. Another interesting property of the bisboronic acid is their ability to bind disaccharides maltose and lactose. For example, the *K*a of **L-2** with α-D-lactose is around 36 M^-1^, while the *K*a of **L-1** is only 1 M^-1^. The most promising finding was the high binding affinity of **D-2** and **L-2** with three tetrasaccharides: neocarratetraose-4^1^-*O*-sulfate, *N*',*N*'',*N*''',*N*''''-tetraacetyl chitotetraose, and lacto-*N*-tetraose (*K*a = 2422~19148 M^-1^). With the high binding of bisamidoboronic acid, there is no significant stereochemical discrimination in the binding for **D-2** and **L-2**. The authors ascribed this to two possible reasons: 1) the side chains did not participate in binding, and 2) no bidentate binding was involved. 

## 7. Copper-Mediated Boronic Acid Degradation and Its Effect on CuAAC

In the presence of a boronic acid compound, it is known that Cu(I) can insert between the carbon-boron bond, which leads to useful coupling reactions when desired [73,74] and degradation as side reactions. For example, in preparing a coumarin-based hydrogen peroxide sensor, which contains a triazole moiety for structural diversification and easy analog synthesis, boronic acid degradation by Cu(I) was a problem. Therefore, the triazole ring had to be introduced before borylation (Scheme 3). In applying this CuAAC reaction to the preparation of boronic acid conjugates, it is important to minimize Cu(I)-mediated decompositions. 

**Figure 2:**
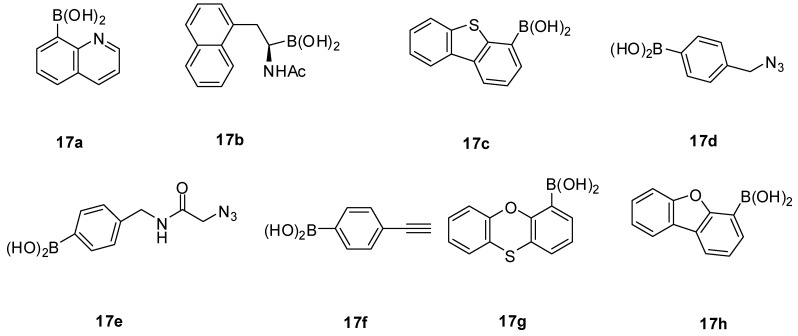
Structures of compounds **17a**~**17h** for the stability study.

The Wang lab recently reported that fluoride was able to protect boronic acids from Cu(I)-mediated decomposition either alone or in a cycloaddition reaction mixture [75]. Such results should be very useful for the future synthesis of boronic acids with diverse structures using CuAAC. In this study, it was found that some boronic acids are less prone to Cu(I)-mediated degradation than others. For example, 8-QBA (**17a**, Figure 2) was essentially stable with 95% remaining after being exposed to 100 mM of Cu(I) for 5 h. On the other hand, for **17e** only 79% of the boronic acid remains under the same conditions. However, addition of fluoride (100 mM) diminished the degradation to a negligible level. Similar results were observed with boronic acids **17f**~**17h**. 

Computational studies indicated that the ability for fluoride to protect arylboronic acids was due to the lowered energy of the fluoride adduct, which makes Cu(I) insertion unfavorable energetically. However, it needs to be pointed out that the ability for fluoride to protect boronic acids from Cu(I)-mediated degradation is not generally applicable to all boronic acids. For example, the degradation of boronic acids **17b**~**17d** was not prevented with the addition of fluoride. The detailed reason why some were not protected is not clear. 

**Scheme 7:**
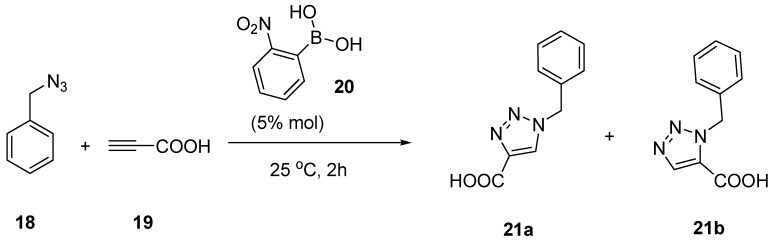
Ortho-nitrophenylboronic acid catalyzed click reaction.

**Scheme 8:**
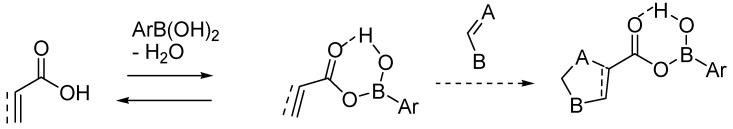
Proposed electrophilic (LUMO-lowering) activation of unsaturated carboxylic acids.

Recently, the Hall lab found that in some cases boronic acids could facilitate the Huisgen cycloaddition in the absence of copper. Specifically, some *ortho*-substituted arylboronic acids have the ability to accelerate the cycloaddition of azides with terminal alkynes attached to a carboxylic group [76]. Among all the boronic acids studied, the best catalysis was observed with 5% ortho-nitrophenyl boronic acid (**20**). At 25 ºC for 2 h, boronic acid **20** was able to catalyze an azido-alkyne cycloaddition with high yield (92%) and regioselctivity (**21a**: **21b **> 98:2) (Scheme 7). The ability for boronic acid to catalyze the cycloaddition reaction is presumably due to the electrophilic (or LUMO-lowering) activation of unsaturated carboxylic acids by the formation of a covalent adduct with a boronic acid group (Scheme 8). The observed catalytic effect of boronic acid is very interesting and useful since it allows for minimization of copper-mediated degradations, though only in cases with certain structural features. 

## 8. Conclusions

During the past decade, interest in and applications of the CuAAC reaction have increased to the point that it permeates all fields in organic, medicinal, and bioorganic chemistry. When applied to boronic acid chemistry, the requirement for a copper catalyst is a limiting factor. Chief among the problems is copper-mediated boronic acid degradation. It has been found that some boronic acids are more prone to copper-mediated degradation than others; fluoride can protect some boronic acids from copper-mediated degradations; and boronic acids in some cases can catalyze the Huisgen cycloaddition reaction. Detailed mechanistic insight into these observations will be very useful to broaden the future application of click chemistry to boronic acid synthesis and conversions. Though alkynes, which can readily undergo copper-free cycloaddition, have been reported [77,78,79], they are not applicable in all situations. Thus CuAAC still has its important place.
